# Mental health outcomes in communities exposed to Armed Conflict Experiences

**DOI:** 10.1186/s40359-021-00626-2

**Published:** 2021-08-27

**Authors:** Sandra Trujillo, Luz Stella Giraldo, José David López, Alberto Acosta, Natalia Trujillo

**Affiliations:** 1 GISAME, Facultad Nacional de Salud Pública, Universidad de Antioquia UdeA, calle 62 Nº 52 - 59, Medellín, Colombia; 2 SISTEMIC, Facultad de Ingeniería, Universidad de Antioquia UdeA, calle 70 No 52-21, Medellín, Colombia; 3 Department of Experimental Psychology, Mind, Brain and Behaviour Research Center (CIMCYC), Universidad de Granada, Granada, Spain; 4 National School of Public Health, University of Antioquia UdeA, Street 62 No. 52–59, Medellín, Colombia

**Keywords:** Mental health, Armed Conflict, Empathy, Extreme Experiences, Anxiety disorders

## Abstract

**Background:**

Populations exposed to Armed Conflict Experiences (ACE) show different levels of impact in their mental health (i.e. clinical and positive components); however, there is limited evidence related to mental health of general population (civilians not classified as victims) exposed to ACE. Government guided mental health assessments exclude this population. The use of a newly validated Extreme Experiences Scale (EX^2^) seems appropriate to classify victims, ex-combatants, and civilians for their mental health assessment.

**Methods:**

Here, we propose a novel approach to identify relationships between individuals classified with different levels of ACE exposure—independent of their legal role in the armed conflict, and mental health outcomes. According to the cut-off points derived from the scores of EX^2^, we classified the sample in low and high exposure to ACE.

**Results:**

The high-level ACE group (scores > 2.5) included 119 subjects, and the low-level ACE was constituted by 66 subjects. Our results evidence that people with high exposure to ACE experiment higher odds to present anxiety disorders, risk of suicide, or post-traumatic stress disorder, as well as increased cognitive empathy (i.e., fantasy dimension).

**Conclusion:**

These findings allowed us to identify the influence of ACE on mental health outcomes beyond the conventional frame (victim or ex-combatant), and to discuss effective interventions and implementation of mental health strategies in these communities. We expect to help the health system to focus on key vulnerable subjects by including civilians not recognized as victims, which are neglected from most of the public health screening, assessment, and interventions.

**Supplementary Information:**

The online version contains supplementary material available at 10.1186/s40359-021-00626-2.

## Background

Armed conflicts are an unfortunate constant of human civilization [1]. Most of the communities exposed to Armed Conflict Experiences (ACE) live in low/middle-income countries with limited resources for social investment and mental health support [2]. Mental health studies among people exposed to ACE have focused on characterizing highly prevalent mental health disorders (e.g. depression, anxiety) and/or their symptoms associated with traumatic ACE, using grouping criteria according to a legal frame (e.g. ex-combatants and victims) [3, 4]. In this sense, Tobón et al. [5] and Sánchez-Padilla et al. [6] characterized adult ex-combatants and victims. They found that 39.9% of such population described distress or anxiety and 39.3% showed sad feelings together with recurrent crying. They also reported diagnosis of depression (18.2%), acute stress disorder (9.9%), and PTSD (8.4%). Additionally, mood and anxiety disorders were larger for those living in rural (46%) than in urban areas (34%) [5, 6]. Moreover, other mental health dispositions such as empathy have also been evaluated. Authors have found that different empathic dispositional profiles (e.g., low empathic concern and personal distress) were observed in ex-combatants when compared to controls based on the Interpersonal Reactivity Index (IRI) assessment [5]. They found that ex-combatants and victims with low empathic scores showed lower neuropsychological rates in working memory and inhibitory control than those with high empathy [5, 6].

Other studies such as the Colombian Mental Health Survey [7] used semi-structured surveys like the Self-Reporting Questionnaire (SRQ) and socio-demographic questionnaires in civilians [8, 9]. They evaluated the associations between exposure to ACE and mental health disorders and revealed that individuals exposed to ACE had a higher probability of showing mental health disorders when compared to non-exposed people [7]. Additionally, other studies identified that civilians exposed to ACE also experienced a higher prevalence of mental health disorders with emotional and psychological affections [8, 10–12].

Colombia is a well-known world referent of a long-term and low-intensity armed conflict with a wide impact on the continent. Official entities for victims such as RUV for its abbreviations I in Spanish (Unique Registry of Victims) inform that 18.5% of Colombian population has been a victim. Reported events were mainly forced displacement (7,553,750), homicide (1,010,989), and harassment (419,229) [13]. Moreover, according to the Colombian Normalization and Reincorporation Agency (ARN: Agencia para la Reincorporación y Normalización), 74,277 people left illegal armed groups between 2001 and 2019 [14]. In addition to these actors (victims and ex-combatants), the Colombian armed conflict has also impacted the general population. The latest mental health survey in the country showed a prevalence of traumatic events related to the armed conflict of 7.7% (95% CI 6.9–8.5) in the general population between 18 and 44 years old [7, 9].

Government programs have prioritized individuals identified as victims or ex-combatants based on the evidence mentioned above. However, there is a limited inclusive analysis of mental health outcomes in civilians exposed to ACE [15]. Thus, related studies include civilians (victims or not) and ex-combatants in a common quantitative category due to the lack of validated measures to control the level of ACE. These situations have blinded the characterization of mental health outcomes in populations exposed to ACE and have constrained the evidence for developing public-health-based screenings, assessments, and interventions focused on reducing the burden of mental health symptoms [15, 16].

Classifying the population according to their level of ACE allows tackling difficulties related to: (a) data quality, by addressing populations at risk with ACE as a measure of exposure; (b) ecological fallacy, by attributing effects that occur at a macro level to individuals [17]; and (c) the influence of ACE in different dimensions (i.e. social, cultural, health) to characterize these events. Quantifying the exposure to ACE will support establishing mental health risks and, therefore, prioritizing key vulnerable groups [18–20].

Previous studies in armed conflict and mental health identified limitations in the reliability of scales and questionnaires aiming to characterize relations between mental health and levels of exposure to ACE [18, 20–22]. There are few instruments validated in Spanish used for this purpose [23]. In this context, in Giraldo et al. [23] we previously validated the Extreme Experiences Scale (EX^2^) with populations exposed to ACE in Colombia. This instrument allowed us to enhance the reliability for classifying individuals according to their ACE in terms of levels of exposure (e.g. low or high). It was sensitive to capture the chronic exposure to ACE expected in scenarios such as the Colombian one. This instrument showed content and face validity, and internal consistency (KR-20: 0.80, 95% CI 0.76–0.84). A two-dimensional factorial structure (direct or indirect exposure to extreme experiences) with an adequate model adjustment was found (CFI 0.91, TLI 0.90, RMSEA 0.05).

In the present study, we aimed to evaluate mental health outcomes in a population with different levels of ACE. Our hypotheses were: (a) EX^2^ will reliably discriminate different levels of ACE in a sample comprised of ex-combatants, victims, and general population (non-victims); and (b) populations with low and high ACE have differential patterns related to mental health outcomes (i.e., post-traumatic stress disorder, other anxiety, and mood disorders, as well as in positive mental health aspects such as variations in empathic dimensions). We expect that this study will contribute with relevant knowledge about the relation between mental health outcomes and ACE, and that it will enhance the attention of mental health services provided to these populations by the local government agencies.

## Methods

### Participants

A sample of 220 adult subjects participated in this study. 35 of them were excluded because of missing data (we excluded subjects if more than 5% of the items on the EX^2^ and IRI scales were not answered). As inclusion criteria, we considered subjects of 18 years old and above who voluntarily agreed to participate. Exclusion criteria were a history of brain damage, use of psychiatric/neurological medication, or substance abuse that may interfere with their ability to complete the questionnaires. All participants were evaluated by a trained psychologist using above mentioned criteria. To reach a heterogeneous sample, we invited different populations with potential exposure to direct or indirect experiences related to the Colombian armed conflict, i.e., we used a convenience sample.

#### Ex-combatants

This sample consisted of 78 Colombian ex-combatants from illegal armed groups (52 men and 26 women; mean age 35.6 years, SD 8.6 years; mean education 9 years, SD 3.6 years) that were active in their reintegration route, provided by the ARN [14]. Each participant was contacted by the ARN professional who oversaw his/her process.

Additionally, we invited participants from the general population and people legally declared as victims. This sample was recruited through an open invitation posted in public schools, governmental institutions, and churches of two selected municipalities of Antioquia, Colombia (see below). We used public schools as meeting points for people interested in participating in the study. Antioquia has been one of the Colombian regions historically most affected by the armed conflict, with actions of different legal and illegal armed actors [24–26]. Moreover, between 2004 and 2009 Antioquia was the second region with higher homicide rates (16,137 homicides) [26]. The two chosen municipalities differed in indicators of displacement, homicides, and actions related to the conflict. This difference aimed to find variability in levels of exposure to ACE. Between 1990 and 2013, in the first municipality, the reported average homicide rate was 125 per 100,000 inhabitants and the average displacement rate was 105.9 per 10,000 inhabitants. For the same period, the second municipality had an average homicide rate of 65.6 per 100,000 inhabitants and an average displacement rate of 25.2 per 10,000 inhabitants [27].

#### Victims

We embraced the status of victim of the armed conflict defined in Law 1448 of the Colombian Constitution [28], in which a victim is someone who suffered an individual or collective damage from events related to armed conflict (i.e., kidnapping, forced disappearance), including his/her permanent companions and first-degree relatives, from January 1, 1985 to present date. This sample consisted of 50 subjects (4 men and 46 women; average age 39.7 years, SD 13.1 years; mean education 10.5 years, SD 3.1 years).

#### General population (civilians not identified as victims)

A group of 57 volunteers that manifested not being exposed to ACE throughout their lives formed this group (18 men and 39 women; average age 35.4 years, SD 15.2 years; mean education 10.8 years, SD 3.6 years). They informed of not having a criminal record and were not directly involved in the armed conflict (either as victims or combatants).

**Fig. 1:**
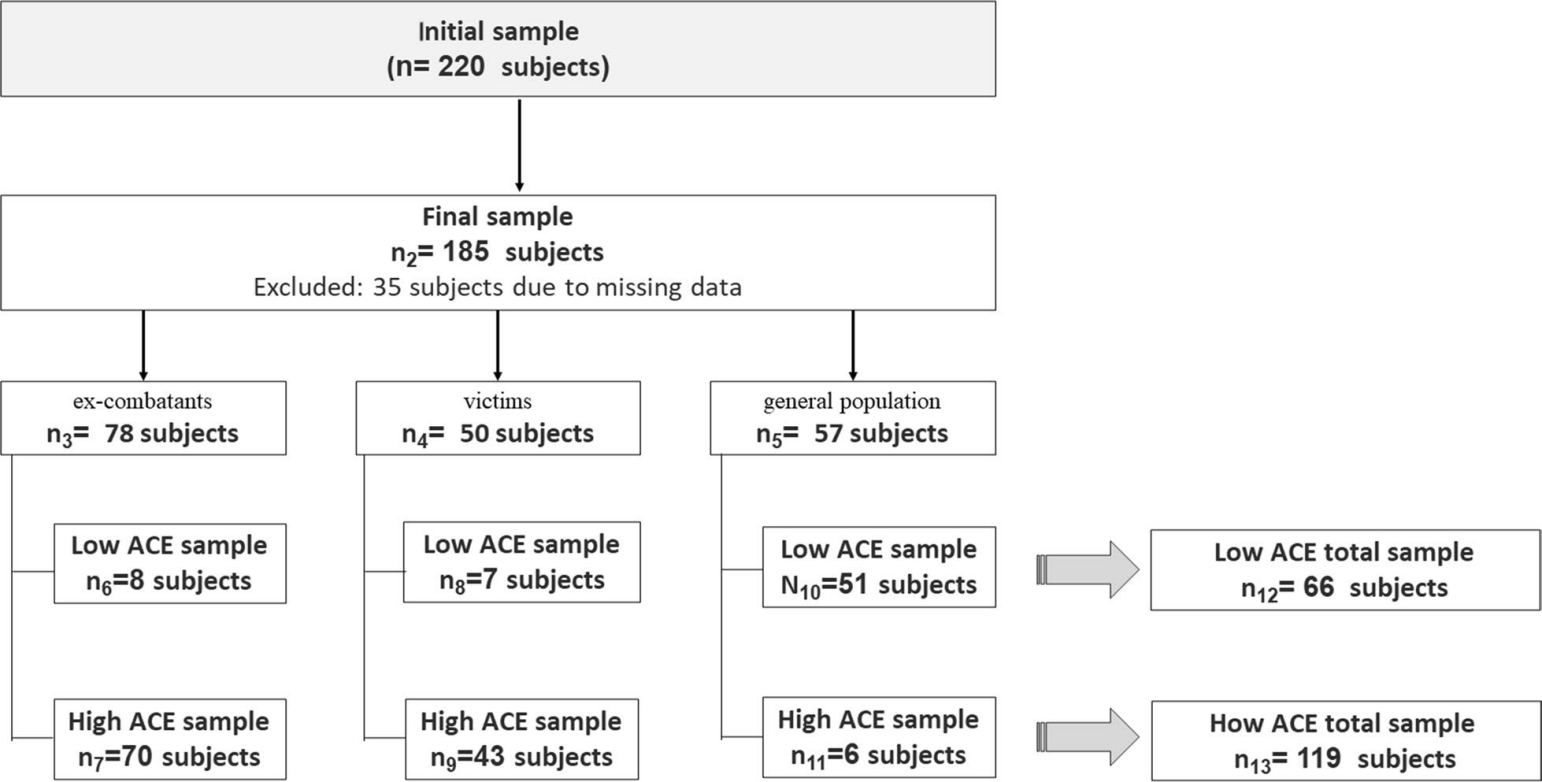
Description of the sample: low and high ACE

Figure 1 presents the sample used in this study. Our final sample consisted of 185 participants that accomplished the minimum sample criterion by item (10 participants per variable) to apply the logistic regression used for the statistical analysis [29, 30]. The participants were divided in two groups: (a) population with low ACE, and (b) population with high ACE. These levels were based on the EX^2^. Scores above 2.5 represent the high exposure group and scores below 2.5 represent low exposure. However, the cut-off point < 2.5 does not indicate absence of exposure. This group includes people who report less than three ACE; that is, they could be exposed to the conflict but to a lesser degree. This cut-off point was previously validated with populations exposed to the Colombian armed conflict (i.e. victims and ex-combatants) [23]. With this sample criterion we were able to evaluate the population according to their ACE level.

Table 1 presents descriptive information of the EX^2^ groups (high and low) for the variables: sex, age, and years of education.

**Table 1:** Demographic information about the groups with ACE

Demographic characteristic	Low ACE (n = 66)	High ACE (n = 119)
	n (%)	n (%)
Sex (*p* value 0.168^a^)		
Women	44 (66.7)	67 (56.3)
Men	22 (33.3)	52 (43.7)
Age (median SD) (*p* value 0.002^b^)	35.2 (14.5)	37.4 (10.8)
Years of education (median SD) (*p* value 0.024^b^)	10.5 (3.7)	9.7 (3.4)

ACE, Armed Conflict Experience

^a^Chi square test of independence

^b^Student *t* test

### Assessment instruments

#### Extreme Experiences Scale (EX^2^)

The EX^2^ is a questionnaire adapted from the Extreme Experiences Inventory [31] and validated for the Colombian armed conflict contexts by Giraldo et al. [23]. It consists of 18 items and two dimensions: (a) direct extreme experiences (e.g., suffering death threats, assaults, or beatings) with 12 items, and (b) indirect extreme experiences with 6 items; that is, those that occur to another person with whom strong coexistence ties are generated, such as family and friends (e.g., murder of a close relative or friend). The EX^2^ scale was a validated to be used in the characterization of extreme experiences in contexts of armed conflicts. The scale has yes/no response options where affirmative answers have a value of 1. The scale score is computed as the sum of its items, it ranges from 0 to 18. The questions inquire if the individual or his/her relatives suffered death threats, aggressions, kidnapping, beating, or any other event considered as exposure to ACE.

The scored 2.5 cut-off point mentioned above was reached through a ROC analysis. Such analysis suggests that individuals under this value experienced less situations associated to armed conflict that those with values higher than 2.5. Ex-combatants and victims commonly have scores over 2.5. In practice, both groups (high vs. low) have exposure to ACE; but this cut-off point is sensitive to discriminating them. Additionally, people with high ACE (score > 2.5) tend to show a higher frequency of events related to the direct dimension [23]. The scale has a good internal consistency (KR-20: 0.80, 95% CI 0.76–0.84) and demonstrated fit for the two-dimensional models (CFI 0.91, TLI 0.90, RMSEA 0.05—IC90% 0.04–0.07). It has a 90% capacity to differentiate individuals between low and high exposure to ACE [23]. With the sample of the present study, we found an excellent reliability of the EX^2^ scale (KR-20 0.80, 95% CI 0.76–0.84).

#### Mini-International Neuro-Psychiatric Interview Version 5.0

The MINI [32] is a structured interview designed to evaluate Axis I diagnoses based on the criteria of DSM–IV [33], assigning a value of 1 for affirmative diagnoses and 0 to the absence of diagnoses. The interview reliability has presented a kappa coefficient ranging from 0.88 to 1.0 and a good test–retest diagnosis, with a kappa between 0.76 and 0.93 [34]. This instrument has been used in populations exposed to war and armed conflicts such as war veterans and refugees [35, 36]. The MINI was used to assess symptoms and mental health diagnoses such as mood disorder, anxiety disorder, alcohol abuse, psychotic disorder, antisocial disorder, PTSD and Suicide risk summarized in Tables 2 and 3.

**Table 2:** Distribution of mental health outcomes by group according to their ACE level

Mental health outcome	Total		Low		High		*p* value
	n	%	n	%	n	%	
Anxiety disorder	41	22.2	9	13.6	32	26.9	0.038^a^
Suicide risk	33	17.8	6	9.1	27	22.7	0.021^a^
Mood disorder	30	16.2	8	12.1	22	18.5	0.260^a^
TEP	19	10.3	2	3.0	17	14.4	0.021^c^
Alcohol abuse	15	8.1	3	4.5	12	10.1	0.263^c^
Psychotic disorder	11	5.9	1	1.5	10	8.4	0.100^c^
Antisocial disorder	7	3.8	1	1.5	6	5.0	0.424^c^
Eating disorder	2	1.1	2	3.0	0	0.0	0.126^c^
Anxiety disorder and mood disorder	17	9.2	4	6.1	13	10.9	0.274^a^
Anxiety disorder and suicide risk	10	5.4	1	1.5	9	7.6	0.099^c^
Mood disorder and suicide risk	11	5.9	3	4.5	8	6.7	0.749^c^
Sample	185		66		119		

ACE, Armed Conflict Experience

**p value < 0.05

^a^Pearson chi square test

^c^Fisher's exact statistic

**Table 3:** Odds ratio associations among variables of mental health outcomes and high/low levels of ACE

	Mood disorder	Anxiety disorder	Alcohol abuse	Psychotic disorder	Antisocial disorder	PTSD	Suicide risk
	OR (CI 95%)	OR (CI 95%)	OR (CI 95%)	OR (CI 95%)	OR (CI 95%)	OR (CI 95%)	OR (CI 95%)
*EX*^*2*^* Scale (ref low ACE)*							
High ACE	1.66 (0.69–4.04)	2.34** (1.02–5.33)	3.19 (0.81–12.63)	5.99 (0.73–49.18)	3.59 (0.39–32.79)	5.63** (1.24–25.67)	3.28** (1.25–8.63)

Adjusted for age, sex, educational level—**p value < 0.05—ACE, Armed Conflict Experience; OR, odds ratio; PTDS, post-traumatic stress disorder

We evaluated the reliability of the MINI in our sample and found a Cronbach's alpha of 0.83 (95% CI 0.79–0.86).

#### Interpersonal Reactivity Index (IRI)

This scale was created by Davis [37], adapted to its Spanish version by Mestre et al. [38], and validated for a sample of Colombian ex-combatants by Garcia et al. [39] and Pineda et al. [40]. This is a 28 item self-report instrument. Nineteen of the items were written in a positive sense and nine in a negative one. It has five-answer options on a Likert scale being the first option “it does not describe me well” and the last one “it describes me very well.” IRI is divided into four dimensions defined as Fantasy (FS), which explores the way that the subject self-identifies with fictional context and characters in stories such as novels, books, or movies; Empathic Concern (EC) evaluates the responses of compassion or sympathy considering misfortunes toward others; Personal Distress (PD) evaluates the response of the subjects under stressor circumstances for themselves and other people; and Perspective Taking (PT), which assesses the ability to consider someone else’s points of view through other people’s experiences and dispositions. The reliability of this scale was validated for Colombian ex-combatants with an Alpha coefficient of 0.76 [39, 40].

For the IRI scale, we found good reliability in our data with a Cronbach's Alpha of 0.72 (95% CI 0.66–0.78).

### Procedure

This is a cross-sectional study that explores the relationship between exposure to ACE in Colombia and mental health outcomes. Initially, participants were informed about the purpose of the study, questionnaires, scales, privacy, confidentiality of data management, and the implications and benefits of participating in this study. Individual acceptance was supported by signing the informed consent. This document was approved by University of Antioquia, (Medellín, Colombia) Medicine School Ethics Committee. Afterwards, they were evaluated by a trained psychologist through an individual interview, in which they were inquired about the presence of neurological or psychiatric conditions and their possible exposure to ACE.

### Statistical analysis

Data analysis started with data cleansing of outliers, missing data, and inconsistent information. For each variable (EX^2^, MINI, and IRI), we accepted a maximum of 5% of missing data and replaced these values with the median; variables above this percentage were eliminated. Only item 28 of the IRI (translated from Spanish: “Before criticizing someone, I try to imagine how I would feel if I were him/her”) surpassed 5% of missing data. It presented 33.5% of missing data and was thus eliminated.

Considering the context of the participants evaluated in this study, we decided to analyze the IRI based on the theoretical model reported by García et al. [39]. To guarantee the reliability of the IRI scale without item 28 and to verify the consistency of the 17 items model, we performed a Confirmatory Factor Analysis (CFA) excluding item 28 to identify changes in the data structure. The analysis suggested that there were no structural changes for the confirmatory model by excluding this item. The complete analysis is available in Additional file 1.

After the preliminary analysis and data cleansing, we evaluated the bivariate association between ACE (obtained with EX^2^) and clinical diagnosis (MINI). We established a chi-square association test. The significance of the analysis was given by a *p*-value < 0.05.

Finally, we explored the EX^2^ level of ACE as a key factor to explain changes in mental health outcomes of populations affected by the armed conflict. To determine the relative weight of ACE, we used a Binary Logistic Regression (BLR) and a Linear Regression Model (LRM), with levels of ACE as the independent variable and mental health outcomes (e.g., depression, anxiety, empathy-fantasy dimension) as dependent variables. We used BLR to analyze clinical diagnoses given the dichotomous nature of the MINI. The outcome variable was 1 (presence of diagnosis) or 0 (absence of a diagnosis), e.g., presence (1) or absence (0) of depression. We used LRM to analyze empathy outcomes given the continuous nature of the IRI. In this case, the outcome variable was the score of each IRI dimension.

Both models were adjusted for demographic variables that might interfere, such as age, years of education, and sex. To observe significant associations in the model BLR between ACE and clinical diagnosis (MINI) outcomes, we estimated the odds ratio with a confidence interval of 95%. To observe significant associations in the model LRM between ACE and empathy outcomes, we estimated coefficients with a confidence interval of 95%.

In both models, the statistical modeling process was carried out using the enter selection method. We used a Hosmer Lemeshow criterion to enter in the multivariate models, variables that passed a bivariate association with a *p*-value > 0.25. For all models, a *p*-value < 0.05 was considered statistically significant. We specified 95% of confidence in the analyses. All the analyses were performed with SPSS 23rd version [41] and Stata 14th version [42].

## Results

Table 2 shows Pearson chi square test or Fisher exact statistic bivariate analyses to explore the associations between ACE levels and the mental health outcomes evaluated in this study. We found significant differences characterized by a large proportion of anxiety disorders (22.2%) in the high ACE group, particularly in PTSD (14.4%) and suicide risk (17.8%). We also evaluated the crossover of some of these diagnoses. Although we did not observe statistical differences, we did find a trend with a high percentage of the following combinations: anxiety disorder and mood disorder (10.9%), anxiety with suicide risk (7.6%), and mood disorder with suicide risk (6.7%).

Table 3 shows the BLR model exploring the relation between exposure to ACE and mental health outcomes evaluated in this study. The BLR analysis was adjusted by age, sex, and years of education. The model explored ACE through the EX^2^ scale and mental health outcomes through the MINI scale.

We found that the group with high ACE had more probability of presenting clinical mental health outcomes. Additionally, the probability to have an anxiety disorder in the sample with high ACE was 2.34 times higher than in subjects with low ACE. Moreover, subjects with high ACE presented higher odds to develop PSTD (OR 5.63; CI 95% 1.24–25.67) and suicide risk (OR 3.28; CI 95% 1.25–8.63).

Table 4 presents the influence of ACE in cognitive and affective dimensions of the IRI scale adjusted by age, sex, and years of education. Results show that a high ACE only influences the Fantasy dimension (β = 1.77, 95% CI 0.28–3.26) when adjusted by other variables in the model. For other cognitive and affective empathy dimensions, ACE did not present significant differences.

**Table 4:** Association between EX^2^ scale and empathy variables from IRI in population exposed to the armed conflict

Lineal model	PT		FS		EC		PD	
	β	CI 95%	β	CI 95%	β	CI 95%	β	CI 95%
*EX*^2^ Scale (ref low ACE)								
High ACE	− 0.82	− 1.75 to 0.08	1.77	0.28 to 3.26	0.71	− 0.18 to 1.60	0.86	− 0.23 to 1.96
Constant	13.12		10.39		10.73		12.20	

According to the Hosmer Lemeshow criterion (*p* < 0.25) all dimensions enter the multivariate model

Adjusted for age, sex, educational level—*p value < 0.05, ACE, Armed Conflict Experience; β, standardized coefficients, CI, coefficient interval; PT, Perspective Taking; FS, Fantasy; EC, empathic concern; PD, personal distress

## Discussion

This study aimed to evaluate mental health outcomes in a sample with different levels of ACE calculated by the EX^2^ scale, a variable that we hypothesized influences the appearance of mental health outcomes (measured by the MINI and the IRI scale). We found that the group with high exposure to ACE presented higher probability of anxiety disorders, PTSD, and suicide risk. Additionally, we found that a high level of ACE explains changes that occur in IRI-fantasia scores, adjusted with age, sex, and educational level, suggesting that this relation is crucial to program the socio-affective response.

Our study was supported by a previous validation of the EX^2^ scale [23]. This allowed us to suggest that the EX^2^ cut-off point of 2.5 is sensitive and reliable to discriminate mental health outcomes according to the low or high level of exposure to ACE. A similar cut-off point was previously reported for traumatic events (not only in armed conflict contexts) by Cherewick et al. [43], where scores for potentially traumatic events were 2.2 and 2.3 for males and females respectively [43]. This may contribute on reducing gaps presented in previous studies that did not classify the level of ACE [18, 20, 22, 23, 44, 45], for example with our finding of a relation between high levels of ACE (through the EX^2^ scale) and mental health disorders (anxiety, PTSD, and suicide risk). Moreover, although the construct of ACE is recent, this finding complements the information reported in other works that evaluated mental health outcomes in different populations exposed to ACE [19, 20, 46, 47] but were focused in only one group (e.g., veterans) or measure (e.g., PTSD).

Regarding the relation of ACE and the IRI scale, our work provides a quantification of the exposure to ACE associated to empathy with similar results than categorical-variable-based studies. Previous research focused on Colombian ex-combatants identified different empathic profiles [48–50]. One of these profiles was effectively characterized by high scores in cognitive dimensions (i.e., FS, PT), suggesting that people exposed to ACE may tend more frequently to assign potential fictional or imaginary explanations to interpret unfortunate situations. Similar results were reported by Agaibi et al. [44], where people exposed to extreme stress and trauma experienced different patterns of coping styles, changing their socio-affective and mental health responses. Empathic dimensions such as fantasy allow creating coping strategies to face traumatic situations in terms of religiosity, or high expectations about how things will get better in a near future. Furthermore, such relation of ACE and fantasy might influence their perception of affective and cognitive states, and the response of their social context as previous studies reported [51].

No other relations were found between ACE and mental health outcomes derived from MINI and IRI. To our knowledge, this is one of the first approaches that relates mental health outcomes (such as clinical conditions and empathy dimensions) with the exposure to ACE. In summary, our study suggests that the EX^2^ cut-off point found in Giraldo et al. [23] may be used as a predictor to explore mental health outcomes (e.g. mental disorders) in people classified with high levels of ACE.

Furthermore, the regression model was relevant to identify the relations between mental health outcomes and different levels of ACE (calculated from the EX^2^ scale). This model advanced in the identification of (a) the influence of the exposure to ACE on the appearance of mental health disorders; and (b) the relation between ACE and changes in empathic dispositions (i.e., fantasy). This model improves the quality of information used to identify risk and protective factors.

A key novelty of our work with respect to previous studies comes from the way that we classified our sample, as other works traditionally use criteria based on a legal framework (i.e. victims, ex-combatants, and refugees) [14, 52]. In this study, we propose a novel analysis of mental health outcomes for individuals exposed to ACE without considering their legal status. Additionally, instead of considering only their mental health diagnosis, we also considered the use of socio-cognitive instruments to evidence social and affective aspects of mental health, such as it is presented in empathy dimensions. We expect that this approach will improve the effectiveness of the attention to screen, assess, intervene, and potentially prevent outcomes in populations affected by these events.

Although our sample size was small when compared to previous studies [6], our statistical model guaranteed: (1) the reliability of the results, because the regression model is a robust model adapted for small sample sizes; (2) we found no differences in socio-demographic variables that commonly work as confounding; and finally, (3) our findings are aligned with previous studies that used larger samples [8, 19, 20]. Additionally, studies on mental health of populations affected by armed conflicts have shown limitations in the reliability of the questionnaires to measure the exposure to ACE [2, 20, 22]. Our study controlled this by using a validated instrument (EX^2^) [23].

The results of our study represent an important piece of evidence for mental health professionals, especially to direct their efforts on strategies oriented to screen, assess, and implement effective interventions required in populations affected by armed conflicts. Moreover, we suggest the importance of considering not only the aspects reported in this study but also other elements of their particular social context (e.g., access to health and educational services). We expect that future studies could develop two lines of actions: (a) to perform a systematic characterization of the samples based on reliable inventories such as EX^2^ in populations affected by ACE; and (b) to implement evidence-based interventions focused on enhancing social abilities, responding to particular contexts and beliefs as reported in previous studies [8, 49, 53]. This would contribute to integrate different approaches such as public health strategies and, therefore, developing cost-effective models to assess mental health risks across populations exposed to ACE.

Such intervention might enhance the sensitivity to evaluate mental health outcomes in armed conflict contexts, providing new evidence to transfer to epidemiological and clinical fields [33, 53]. We envisage that the replication of our results will inform mental health public policies adapted to populations exposed to ACE. We expect that future studies will promote the use and transference of these associative models, not only to communities chronically exposed to armed conflicts but also populations with extreme vulnerability experiences, such as refugees and people affected by forced displacement. Additionally, we expect further advances in the study of mental health outcomes and coping strategies observed in populations exposed to ACE [10].

## Conclusion

This is one of the first studies focused on classifying people exposed to ACE in terms of low or high levels of exposure and establishing an association with mental health outcomes such as anxiety, risk of suicide, PTSD, and fantasy. The EX^2^ is one of the first instruments, which allow classifying populations based on the exposure to ACE, avoiding the use of legal labels (e.g., victim, military, or ex-combatant). It contributes with new evidence to improve characterization and potential evidence-based intervention programs.

## Supplementary Information

**Additional file 1**. Confirmatory factor analysis of IRI- short version based on Garcia-Barrera et al (39).

## Data Availability

The datasets used and/or analyzed during the current study are available from the corresponding author on reasonable request.

## Abbreviations

ACE: Armed Conflict Experience
ARN: Colombian Normalization and Reincorporation Agency (Agencia para la Reincorporación y Normalización)
BLR: Binary logistic regression
EC: Empathic concern
EX^2^: Extreme Experience Scale
FS: Fantasy
IRI: Interpersonal Reactivity Index
MINI: Mini-International Neuropsychiatric Interview Version 5.0
PD: Personal distress
PT: Perspective Taking
PTSD: Post-traumatic stress disorder
RUV: Sole Registration of Victims
SRQ: Self Reporting Questionnaire
WHO: World Health Organization
