# Supplementary material for: Mental health outcomes in communities exposed to Armed Conflict Experiences
Source: BMC Psychol. 2021 Aug 27;9:127. doi: 10.1186/s40359-021-00626-2 (PMC8394205; doi:10.1186/s40359-021-00626-2)
Supplement: Supplementary file 1 — Additional file 1. Confirmatory factor analysis of IRI- short version based on Garcia-Barrera et al (39). [file 40359_2021_626_MOESM1_ESM.docx]

**Supplementary Material 1.**

**Mental Health Outcomes in Communities Exposed to Armed Conflict Experiences**

**Sandra Trujillo^1^; Luz Stella Giraldo^1^; José David López^2^; Alberto Acosta^3^; Natalia Trujillo^1^**

1. GISAME, Facultad Nacional de Salud Pública, Universidad de Antioquia UdeA, calle 62 Nº 52 - 59, Medellín, Colombia.
2. SISTEMIC, Facultad de Ingeniería, Universidad de Antioquia UdeA, calle 70 No 52-21, Medellín, Colombia
3. Department of Experimental Psychology, Mind, Brain and Behaviour Research Center (CIMCYC), Universidad de Granada, Granada, Spain.

**Confirmatory factor analysis of IRI - García et al model (1)**

Considering the context of the participants evaluated in this study, we decided to analyze the IRI based on the theoretical model reported by García et al. (1). To guarantee the reliability of the IRI scale without item 28 and to verify the consistency of the 17 items model, we performed a Confirmatory Factor Analysis (CFA) excluding item 28 to identify changes in the data structure. The analysis suggested that there were no structural changes for the confirmatory model by excluding this item.

We proceeded to analyze the structure validity by means of Confirmatory Factorial Analysis (CFA) for four dimensions according to the model’s García et al. (1): Fantasy (FS), Empathic Concern (EC), Personal Distress (PD) and Perspective Taking (PT). The models were built by estimating structural equation models of the Stata version 14 (2).

In order to test goodness-to-fit for the model, we relied on chi-square test, comparative fit indexes (CFI), the Tucker–Lewis index (TLI), and the Root Mean Square Error of Approximation (RMSEA). Guidelines proposed (3) suggest that models with CFI and TLI close to 0.90 or higher, RMSEA between 0.05 and 0.08 are representative of good fitting.

**Results**

The confirmatory analysis for the 17 items model (see Figure 1 and table 1) reported a good fit of the empirical data with the theoretical four-dimensional model. The CFI, TLI, and CD indicators were higher at 0.94, *X^2^* was 0.05, RMSEA was 0.04. For the IRI scale, we found good reliability in our data with a Cronbach's Alpha of 0.72 (95% CI, 0.66 - 0.78).

**Table 1.** Data analysis of construct validity and Confirmatory Factor Analysis - IRI

| **Indicators** | **Model 17 Items** |
| --- | --- |
| X^2^ | 0.052 |
| CFI | 0.96 |
| TLI | 0.95 |
| CD | 0.99 |
| RMSEA | 0.33 (CI 90% 0.00 – 0.51) |
| SRMR | 0.062 |


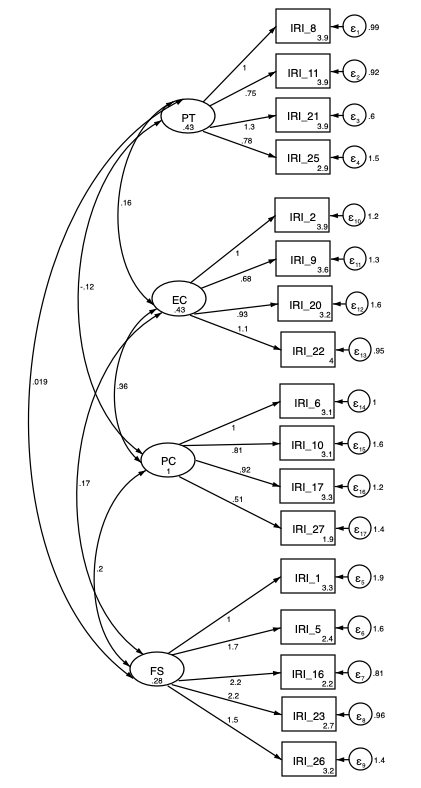


**Figure 1.** Factor structure of the *IRI* validated by García et al(1) without item 28

**Reference**

1. Garcia-Barrera MA, Karr JE, Trujillo-Orrego N, Trujillo-Orrego S, Pineda DA. Evaluating empathy in Colombian ex-combatants: Examination of the internal structure of the Interpersonal Reactivity Index (IRI) in Spanish. Psychol Assess. 2017;29(1):116-22.

2. StataCorp LLC. Stata user guide release 14. Texas: DtataCorp LLC; 2015.

3. Hu L, Bentler P. Cutoff criteria for fit indexes in covariance structure analysis: Conventional criteria versus new alternatives. Structural Equation Modeling: A Multidisciplinary Journal. 1999;6(1):1-55.
